# Use of Anisotropy, 3D Segmented Atlas, and Computational Analysis to Identify Gray Matter Subcortical Lesions Common to Concussive Injury from Different Sites on the Cortex

**DOI:** 10.1371/journal.pone.0125748

**Published:** 2015-05-08

**Authors:** Praveen Kulkarni, William Kenkel, Seth P. Finklestein, Thomas M. Barchet, JingMei Ren, Mathew Davenport, Martha E. Shenton, Zora Kikinis, Mark Nedelman, Craig F. Ferris

**Affiliations:** 1 Northeastern University, Boston, Massachusetts, United States of America; 2 Biotrofix, Waltham, Massachusetts, United States of America; 3 Brigham and Women's Hospital, Boston, Massachusetts, United States of America; 4 Ekam Imaging, Boston, Massachusetts, United States of America; University of Pennsylvania, UNITED STATES

## Abstract

Traumatic brain injury (TBI) can occur anywhere along the cortical mantel. While the cortical contusions may be random and disparate in their locations, the clinical outcomes are often similar and difficult to explain. Thus a question that arises is, do concussions at different sites on the cortex affect similar subcortical brain regions? To address this question we used a fluid percussion model to concuss the right caudal or rostral cortices in rats. Five days later, diffusion tensor MRI data were acquired for indices of anisotropy (IA) for use in a novel method of analysis to detect changes in gray matter microarchitecture. IA values from over 20,000 voxels were registered into a 3D segmented, annotated rat atlas covering 150 brain areas. Comparisons between left and right hemispheres revealed a small population of subcortical sites with altered IA values. Rostral and caudal concussions were of striking similarity in the impacted subcortical locations, particularly the central nucleus of the amygdala, laterodorsal thalamus, and hippocampal complex. Subsequent immunohistochemical analysis of these sites showed significant neuroinflammation. This study presents three significant findings that advance our understanding and evaluation of TBI: 1) the introduction of a new method to identify highly localized disturbances in discrete gray matter, subcortical brain nuclei without postmortem histology, 2) the use of this method to demonstrate that separate injuries to the rostral and caudal cortex produce the same subcortical, disturbances, and 3) the central nucleus of the amygdala, critical in the regulation of emotion, is vulnerable to concussion.

## Introduction

An estimated 1.7 million people in the United States sustain a traumatic brain injury (TBI) each year. There are enormous costs associated with these injuries-52,000 deaths, 275,000 hospitalizations, and ca. 1.4 million individuals treated and released from emergency departments. TBI is a contributing factor to a third of all injury-related deaths in the United States. Among military personnel, there are over 30,000 medically diagnosed cases of TBI annually. There are no known treatments to reverse or minimize the initial brain damage caused by moderate to severe TBI, which represents a critical gap in care. Health services focus on rehabilitation in the areas of physical therapy and speech/language therapy. Psychiatric intervention is often necessary to control the anxiety, depression, and PTSD like symptoms.

Advances in medical imaging, particularly in the field of magnetic resonance imaging (MRI) have been critical in the evaluation of brain injury and disease progression following TBI [1]. MRI can identify the edematous tissue at the site of contusion, global gray matter and white matter damage and hemorrhagic lesions. MRI can be used to evaluate long-term volumetric changes in brain areas that can be correlated with neuropsychological tests of motor and cognitive function. Diffuse axonal injury is the underlying neuropathology common to all TBI and disruptions in connectivity between integrated neural networks affect cognitive and emotional behavior. Diffusion tensor imaging (DTI) and quantitative anisotropy, which indicate the integrity of white matter, can identify subtle changes in the diffusion of water thereby assisting in the identification of specific areas of axonal injury following TBI (e.g., [2]. Different diffusion indices of anisotropy in gray matter can also be used as biomarkers of disease progression such as changes in apparent diffusion coefficient (ADC) in severe TBI [3] and in fractional anisotropy (FA) in aging [4] and therefore, changes in diffusion indices have the potential to evaluate the efficacy of therapeutic interventions *in vivo*. While clinical studies report changes in indices of anisotropy, how these changes relate to disease progression is unknown. A decreased FA is possibly a sign of inflammation [5] and better prognosis of a good outcome. Generally increased RD and AD are indicative of change for the worse. Increased FA, shortly after injury, might have poorer outcome [6, 7]

Can different indices of anisotropy be used to identify subtle changes in gray matter? Indeed, recent studies reported changes in neocortical brain regions in ADC in severe TBI and multiple sclerosis [3, 8] and in FA in aging [4]. Measurements of radial diffusivity (RD) and axial diffusivity (AD), in addition to FA, were reported for patients with persistent post-concussive symptoms [9]. Changes of AD versus changes of RD were analyzed for each individual patient and two different patterns were found. One pattern is defined by decrease in both the AD and the RD, and the other pattern by increased AD with little or no change in RD. The latter pattern suggests gliosis as the underlying pathological process and has been originally established in an animal model [10]. In summary, these studies advocate for the AD and RD to be reported along with FA in studies of brain gray matter.

Animal models of TBI provide opportunities not possible in human studies. Researchers can design prospective, longitudinal studies using histological methods with timed sacrifice to evaluate the molecular and cellular events of disease progression. With the advent of MRI, the same imaging modalities used in humans can be applied to preclinical studies of TBI. Nonetheless, areas identified with MRI as possible sites of subtle gray matter injury must be validated with post-mortem histology. To this end, we have developed analytical methods using a 3D segmented and annotated rat atlas and quantitative anisotropy to identify sites of putative gray matter injury from over 150 brain areas following cortical contusion. We demonstrate in this study that fluid percussion injury (FPI) in the rat at two separate sites on the cortical mantel share common subcortical brain nuclei whereby significant differences in diffusivity are evident from the contralateral side of the brain. With post-mortem histology we are able to focus our efforts on these areas, where we are able to confirm the presence of both neuroinflammation and axonal damage.

## Materials and Methods

### Animals

Adult male Sprague Dawley rats (n = 13) weighing ca.300–350 g each were obtained from Biotrofix (Waltham, MA), a contract research organization that specializes in neurological models for preclinical research. Each animal was subjected to TBI using the fluid percussion model. The fluid percussion model can be adjusted to deliver a force to the brain that reproducibly causes mild to moderate TBI with neuropathological, cognitive and behavioral consequences that reflect those seen in human head injury [11]. Following surgery, animals were housed at Biotrofix for five days, then moved to Northeastern University, Center for Translational NeuroImaging in the early morning, and imaged while alive the same day. Animals were acquired and cared for in accordance with the guidelines published in the Guide for the Care and Use of Laboratory Animals (National Institutes of Health Publications No. 85–23, Revised 1985). These studies were approved by the Biotrofix and Northeastern University Institutional use and Animal Care Committees. Following imaging all animals were euthanized by carbon dioxide followed by thoracotomy.

### Surgery

Animals were anesthetized with 1–3% isoflurane in a mixture of nitrous oxide and oxygen (2:1). The skin at the site of surgery was shaved and the animal then placed in a stereotaxic frame. The skull was exposed through a midline incision. A parasagittal craniotomy (5 mm diameter) using a trephine drill was performed at 1 mm anterior and 1 mm lateral to bregma (n = 5) for rostral concussions and 3.8 mm posterior and 2.5 mm lateral to the midline (n = 8) for caudal concussions. A sterile plastic injury tube was next placed over the exposed dura, bonded and secured. On the next day, animals were re-anesthetized then connected to the fluid percussion device. The device consists of a Plexiglas cylindrical reservoir bounded at one end by a rubber-covered Plexiglas piston and the opposite end fitted with a transducer housing and a central injury screw adapted for the animal’s skull. The injury was induced by the descent of a metal pendulum striking the piston, thereby injecting a small volume of sterile saline into the closed cranial cavity and producing a brief displacement of neural tissue. The amplitude of the resulting pressure pulse was measured in atmospheres by a pressure transducer (2.5–3.0 atmospheres). This procedure produces a moderate TBI [12] characterized by a focal brain contusion and cell death under the point of the connector tube on the dura and a percussion wave through the brain that causes focal white matter damage and neuronal death in the distant hippocampus. The brain injury results in focal motor deficits in the opposite limbs and memory disturbances. There is no mortality during post-TBI. It should be noted the contralateral side was not exposed to sham surgery. Previous studies in our laboratory showed no visible injury to the brain as determined by histology.

### Anatomical Scans

**Fig 1:**
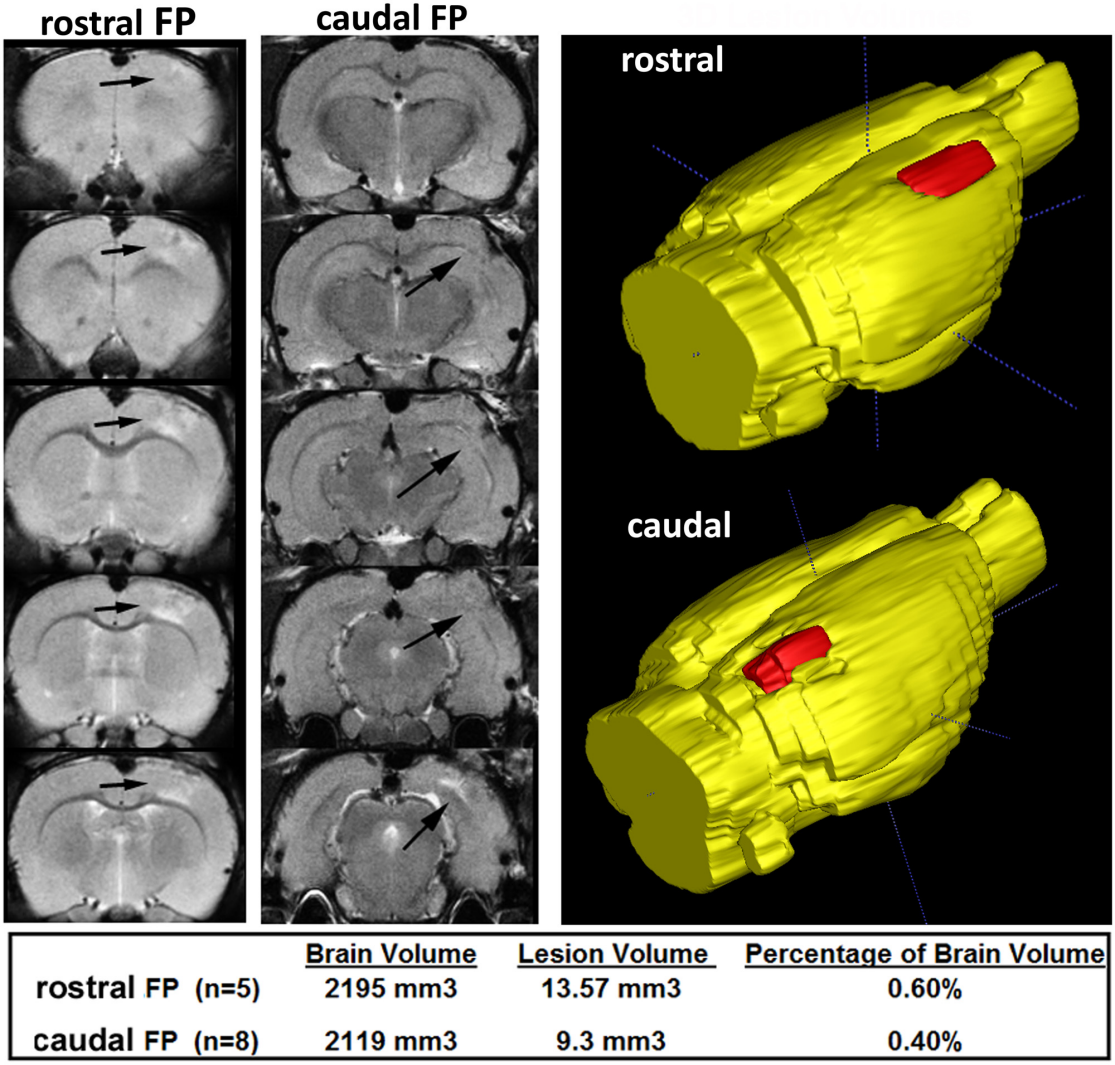
Contusion Location and Volume. Shown in the two left columns are contiguous axial sections of representative rats that received fluid percussion injury in the rostral or caudal cortex. The sections cover the caudal/rostral boundaries of the contusion as indicated by the arrows. The location and volume of the lesion for each contusion site are shown in red in the 3D yellow reconstruction of the full brain on the right. The lesion volume can be calculated from seeding and thresholding the 2D lesion followed by segmentation and volume rendering over the entire lesion from which measures can be taken for statistical analysis. The table below reports the average brain volume, average lesion volume and the percentage of the lesion volume to whole brain volume for both the rostral and caudal injuries.

Experiments were conducted using a Bruker Biospec 7.0T/20-cm USR horizontal magnet (Bruker, Billerica, Massachusetts) and a 20-G/cm magnetic field gradient insert (ID = 12 cm) capable of a 120-μs rise time (Bruker). Radiofrequency signals were sent and received with a quadrature volume coil built into the animal restrainer (Animal Imaging Research, Massachusetts, USA). At the beginning of each imaging session, a high-resolution anatomical data set was collected using the RARE pulse sequence (20 slice; 1 mm; field of vision [FOV] 3.0 cm; 256 × 256; repetition time [TR] 2.5 sec; echo time [TE] 12.4 msec; NEX 6; 6.5-minute acquisition time). An example of anatomical images is provided in Fig 1. These anatomical images are not only used to visualize the position and extent of the cortical lesion, they are also necessary for registration of the data collected from the other imaging modalities.

### T2 Relaximetry

Images were acquired using a multi-slice multi-echo (MSME) pulse sequence. The echo time (TE) was 11 ms, and 16 echoes were acquired during imaging with a recovery time (TR) of 2500 ms. Images were acquired with a field of view [FOV] 3 cm^2^, data matrix = 256×256×20 slices, thickness = 1 mm. Values for longitudinal relaxation time (T_2_) were obtained from all the slices using ParaVison 5.1 software. T_2_ was used to characterize the site of injury on the cortex. No significant changes were found except at the point of injury where edema was present. The T_2_ values were used for segmentation of the area of edema and to quantify the volume of the injured site. The T_2_ values were computed using the equation; y = A+C*exp (-t/T2) (S.D. weighted) obtained from the Paravision 5.1 software. Where, A = absolute bias, C = signal intensity, t = echo time and T2 = spin-spin relaxation time. The edematous tissue was identified as a hyperintensity on the T2 map on the affected ipsilateral side of the brain. The lesion volume was calculated using a snake region growth algorithm in itk-SNAP (www.itksnap.org). The threshold was set at 6300 to 9000 as absolute pixel intensity. A point is seeded within the edema region and the algorithm run until segmentation is complete [13].

### Diffusion Tensor Imaging

Quantitative MRI reflects the physical properties of proton spins in water that create the contrast that characterize the properties of different tissue. These measures include fractional anisotropy (FA) and apparent diffusion coefficient (ADC) or mean diffusivity. The determinants of indices of diffusion at a microscopic level are many as the microarchitecture of the brain parenchyma is composed of neurons and their axonal and dendritic fibers, glia, connective tissue, capillaries, and intracellular and extracellular water. At a macroscopic level, the coherence of the axons in a voxel, i.e., are they parallel or crossing, is the key determinant. It should be noted that microscopic axonal properties and general microarchitecture of a voxel together with fiber coherence are the key determinants of diffusion anisotropy and not myelination as originally posited [14]. Additional indices of diffusion are provided by measures of radial diffusivity (RD), which is the diffusivity in directions perpendicular to the principal axis of diffusion (i.e. (***λ***2 + ***λ***3)/2) and axial diffusivity (AD), which is the diffusivity along the principal axis (i.e. ***λ***1). Work by Song and colleagues indicate that RD is a measure of myelin while AD is a measure of axonal integrity [15]. Accordingly, diffusion tensor imaging is a very sensitive method that can detect subtle micro- and macrostructural changes in tissues, but cannot specify what changes occurred. The specificity of the changes is better addressed by histological methods.

### Pulse Sequences

DTI was acquired with a diffusion-weighted (DW) spin-echo echo-planar-imaging (EPI) pulse sequence having the following parameters: TR/TE = 500/20 ms, eight EPI segments, and 10 non-collinear gradient directions with a single b-value shell at 1000 s/mm^2^ and one image with a b-value of 0 s/mm^2^ (referred to as b0). Geometrical parameters were: 60 slices, each 0.313 mm thick (brain volume) and with in-plane resolution of 0.313×0.313 mm^2^ (matrix size 96×96; FOV 30 mm^2^). The imaging protocol was repeated two times for signal averaging. Each DTI acquisition took 44 min and the entire MRI protocol lasted about 1 hour 28 min.

### Image Analysis

Image analysis included DTI analysis of the DW-3D-EPI images to produce the FA, AD and RD maps. DTI analysis was implemented with Matlab (The Mathworks, Inc. USA) and MedINRIA (1.9.0; http://www-sop.inria.fr/asclepios/software/MedINRIA/index.php) software. Because sporadic excessive breathing during DTI acquisition can lead to significant image motion artifacts that are apparent only in the slices sampled when motion occurred, each image (for each slice and each gradient direction) was automatically screened, prior to DTI analysis, for motion artifacts. Following the elimination of acquisition points with motion artifacts, the remaining acquisition points were corrected for linear (motion) and non-linear (eddy currents/susceptibility) artifacts using SPM8 (Welcome Trust Centre for Neuroimaging, London, UK).

For statistical comparisons between rats, each brain volume was registered with the 3D rat atlas allowing voxel-based statistics [16, 17]. All image transformations and statistical analyses were carried out using in-house MIVA software [18, 19] with affine registration. For each rat, the b0 image was co-registered with the b0 template (using a 6-parameter rigid-body transformation). The co-registration parameters were then applied on the different DTI indexed maps (FA, AD, RD). Normalization was performed on the maps since they provide the most detailed visualization of brain structures and allow for more accurate normalization. The normalization parameters were then applied to all DTI indexed maps. The normalized indexed maps were smoothed with a 0.3-mm Gaussian kernel. To ensure that FA values were not affected significantly by the pre-processing steps, we used the ‘nearest neighbor’ option following registration and normalization.

One of the major advantages to animal imaging is the homogeneity of the subject pool. Commercial, inbreed strains of rats are essentially genetically identical. Using male rats of the same weight and age, as in this study, assures that the size and shape of the brains are indistinguishable. When registered into a 3D segmented, annotated rat atlas, the anatomical fidelity across subjects is highly conserved. In this study ca 20,000 isotropic voxels with one of five different values of IA were localized to one of 150 discrete 3D brain volumes. In the case of the central nucleus of the amygdala, ca 46 voxels occupy this volume, equally divided between left and right sides. The variation between voxel numbers for brain area between subjects is usually less than 2%. In this study the variance in the mean IA values was extremely small where the SD was less than 10% in a group of only five animals. This made it possible to compare 150 bilateral brain areas for difference in IA values using a small sample of animals.

### Histology

Immediately after imaging, five days post-concussion, rats from the rostral cortical TBI group were deeply anesthetized and perfused with 4% paraformaldehyde via cardiac puncture. Brains were then extracted and left for fixation for 24 hours at 4°C in 4% paraformaldehyde. Subsequently, brains were sunk in 20% sucrose for 24 hours for cryogenic protection of tissue prior to sectioning on a cryostat. Brain tissue was then coronally sectioned on a freezing microtome at 40 μm.

Tissue was processed for single label immunohistochemistry according to previously published methods, with the following modifications: a 0.3% hydrogen peroxide was substituted for the phenylhydrazine step and primary antibodies were selected to examine axonal integrity and astrocyte activation. Serial sets (every 3^rd^ section) of free-floating tissue sections were rinsed in 0.05 M potassium phosphate buffered saline (KPBS) to remove excess cryoprotectant. Sections next were incubated in 1% sodium borohydride for 20 min at room temperature to reduce free aldehydes to alcohol followed by a rinse in KPBS. Tissue was incubated in primary antibodies for either glial fibrillary acidic protein (GFAP, 1:1500, AbCam ab7260) or myelin basic protein (MBP, 1:1000, AbCam ab40390) for 1 hour at room temperature followed by 48 hours at 4°C. Sections were then rinsed in KPBS before being incubated for 1 hour at room temperature in biotinylated goat, anti-rabbit IgG (Vector Labs; 1:600 dilution in KPBS + 0.4% Triton X-100). Sections were rinsed again in KPBS and then incubated in an avidin-biotin peroxidase complex (45μl A, 45 μl B per 10 ml KPBS + 0.4% Triton X-100; Vectastain ABC kit-elite pk-6100 standard; Vector Labs) for 1 h at room temperature. Sections were rinsed in KPBS and then with tris buffered saline. Immunoreactivity was visualized by incubation in a solution containing 50 ml of tris buffered saline, 1.25 g nickel sulfate, 41.5 μl of 3% H_2_O_2_ and 10 mg of diaminobenzidine for 15 min at room temperature. Sections were rinsed in tris buffered saline following by a series of rinses in KPBS.

Sections were mounted on gelatin-coated slides and air-dried overnight. Sections were then dehydrated in ascending ethanol dilutions and cleared with Histoclear (National Diagnostics). Slides were then cover slipped with Histomount (National Diagnostics). Images were acquired using a Nikon Eclipse E 800 microscope with 2x and 4x objectives giving 20x and 40x magnification, respectively and a Sensi-cam camera, and IP Lab 3.7 computer software (Scanalytics Inc., Fairfax, VA). All camera/microscopy parameters were standardized; measurement of optic density was standardized, but the OD results were not normalized or transformed. Immunohistochemical staining was quantified in the central nucleus of the amygdala, the hilus of the hippocampus and the laterodorsal thalamus. Three slices were imaged and quantified for each brain region. Staining was measured when it exceeded a threshold pixel intensity of approximately twice background using ImageJ (NIH) and compared between the concussed hemisphere and the non-concussed contralateral hemisphere within each animal. Optical density was measured as the number of pixels above threshold within a brain region, each of which were of a standardized size. Immunohistochemical data were compared via two-tailed paired t-tests for each brain region and stain.

## Results

Representative examples of FPI from two rats, one with a rostral cortical contusion and the other with a caudal contusion, are shown in Fig 1. The contiguous axial brain sections show the deformation of cortical tissue and change in tissue contrast using a T2 weighted imaging protocol. The 3D reconstructions of the brain show the calculated lesion volume in red for each cortical insult. The average lesion volumes for rostral cortex (n = 5) and caudal cortex (n = 8) ranged between 9–14 mm^3^ and qualifies these TBIs as mild to moderate [12].

Table 1 shows the comparison in FA values between the concussed (ipsilateral) and non-concussed (contralateral) sides of the brain from rostral and caudal injury, for different regions of interest, rank ordered for their significance, taken from 150 discrete areas in the 3D segmented rat atlas. Tables 1–3 report only those brain areas that were significantly different between ipsilateral and contralateral sides. The complete statistical summary for all 150 brain areas, for all IA values, and for rostral and caudal concussions are available through the PLOS One data repository. The general trend in these brain areas is an increase in FA on the concussed ipsilateral side of the brain, particularly the amygdaloid complex, and hippocampal areas. However, there are a few exceptions. In the rostral cortical concussions these include the laterodorsal thalamus and lateral geniculate. In the caudal concussions these include the lateral and basal amygdala, anterior cingulate and agranular insular cortices, paraventricular nucleus and laterodorsal thalamus. Areas underlined are common to both rostral and caudal concussions. The direction of the IA values were consistent between rostral and caudal insults (Tables 1–3). For example, if the FA value for the central nucleus of the amygdala was greater on the ipsilateral side than the contralateral side following rostral cortical concussion (0.582 > 0.414), it was also greater following caudal concussion (0.419 > 0.320). The only exception was the lateral amygdala for FA values.

**Table 1:** Fractional Anisotropy.

Right Rostral Cortical Insult—FA Values					
Region of Interest(ROI)	contralateral (left)		ipsilateral (right)		
	FA values	SD	FA values	SD	t-test
CA1 dorsal hippocampus	0.336	0.021	0.410 ↑	0.010	0.001
central amygdala	0.414	0.044	0.582 ↑	0.033	0.001
medial amygdala	0.482	0.059	0.618 ↑	0.013	0.001
laterodorsal thalamus	0.436	0.068	0.276 ↓	0.042	0.002
dorsomedial striatum	0.368	0.018	0.414 ↑	0.017	0.003
lateral geniculate	0.316	0.024	0.278 ↓	0.011	0.012
somatosensory ctx secondary	0.220	0.012	0.246 ↑	0.013	0.013
lateral amygdala	0.262	0.036	0.356 ↑	0.055	0.013
subiculum hippocampus	0.298	0.033	0.344 ↑	0.015	0.021
nucleus brachium	0.208	0.042	0.272 ↑	0.039	0.046
Right Caudal Cortical Insult—FA Values					
Region of Interest (ROI)	contralateral (left)		ipsilateral (right)		
	FA values	SD	FA values	SD	t-test
laterodorsal thalamus	0.488	0.050	0.357 ↓	0.060	0.001
dentate gyrus hippocampus	0.203	0.016	0.245 ↑	0.021	0.001
CA3 hippocampus ventral	0.241	0.046	0.343 ↑	0.047	0.001
globus pallidus	0.356	0.047	0.457 ↑	0.047	0.001
auditory ctx	0.173	0.031	0.221 ↑	0.024	0.003
basal amygdala	0.264	0.014	0.232 ↓	0.021	0.004
somaotsensory ctx secondary	0.186	0.021	0.216 ↑	0.016	0.007
somatosensory ctx primary	0.208	0.028	0.242 ↑	0.014	0.007
temporal ctx	0.196	0.023	0.230 ↑	0.021	0.008
insular ctx	0.199	0.019	0.226 ↑	0.018	0.011
gustatory ctx	0.250	0.028	0.288 ↑	0.026	0.013
paraventricular thalamic nuclei	0.353	0.037	0.301 ↓	0.036	0.013
central amygdala	0.320	0.053	0.418 ↑	0.083	0.013
medial geniculate	0.260	0.025	0.288 ↑	0.017	0.018
medial septum	0.356	0.037	0.400 ↑	0.028	0.019
lateral amygdala	0.256	0.030	0.210 ↓	0.040	0.020
infralimbic ctx	0.288	0.047	0.232 ↓	0.040	0.023
agranular insular ctx	0.225	0.019	0.252 ↑	0.025	0.025
anterior cingulate ctx	0.276	0.037	0.232 ↓	0.034	0.026
cortical amygdala	0.256	0.034	0.300 ↑	0.037	0.026

Measures of fractional anisotropy (FA) following lateral fluid percussion injury to the rostral (n = 5) and caudal (n = 8) cortex. Statistical differences between 150 brain regions comparing FA values between the affected ipsilateral side (right) and the contralateral side (left) are rank order for significance. Values are presented as the mean and standard deviation (SD). Areas underlined are common to both sites of cortical injury. Arrows denote the direction of the difference between the affected and control sides. Note that there is predominately an increase in FA with a few exceptions e.g., laterodorsal thalamus, basal amygdala, infralimbic ctx. Note the lateral amygdala shows a change in direction in FA values between rostral and caudal concussions.

**Table 2:** Radial Diffusivity.

Right Rostral Cortical Insult—RD Values					
Region of Interest(ROI)	contralateral (left)		ipsilateral (right)		
	RD values	SD	RD values	SD	t-test
CA1 dorsal hippocampus	0.256	0.022	0.330 ↑	0.007	0.001
central amygdala	0.316	0.033	0.492 ↑	0.044	0.001
medial amygdala	0.394	0.053	0.540 ↑	0.014	0.001
laterodorsal thalamus	0.388	0.073	0.210 ↓	0.035	0.001
dorsomedial striatum	0.284	0.015	0.340 ↑	0.024	0.002
subiculum hippocampus	0.232	0.029	0.286 ↑	0.018	0.008
lateral amygdala	0.204	0.027	0.310 ↑	0.063	0.009
cochlear nucleus	0.212	0.028	0.316 ↑	0.081	0.026
lateral geniculate	0.234	0.026	0.200 ↓	0.012	0.030
nucleus brachium	0.180	0.027	0.224 ↑	0.032	0.048
Right Caudal Cortical Insult—RD Values					
Region of Interest(ROI)	contralateral (left)		ipsilateral (right)		
	RD values	SD	RD values	SD	t-test
dentate gyrus hippocampus	0.168	0.012	0.207 ↑	0.018	0.001
laterodorsal thalamus	0.434	0.061	0.298 ↓	0.064	0.001
CA3 ventral hippocampus	0.193	0.041	0.286 ↑	0.048	0.001
globus pallidus	0.265	0.039	0.357 ↑	0.054	0.002
auditory ctx	0.126	0.023	0.162 ↑	0.015	0.002
somatosensory ctx secondary	0.134	0.016	0.158 ↑	0.011	0.003
gustatory ctx	0.190	0.017	0.220 ↑	0.021	0.007
paraventricular thalamic nuclei	0.326	0.024	0.286 ↓	0.027	0.007
insular ctx	0.145	0.014	0.166 ↑	0.013	0.007
central amygdala	0.245	0.038	0.340 ↑	0.078	0.008
somatosensory ctx primary	0.158	0.018	0.180 ↑	0.013	0.011
cortical amygdala	0.198	0.029	0.252 ↑	0.045	0.012
temporal ctx	0.145	0.021	0.171 ↑	0.015	0.012
medial septum	0.273	0.034	0.315 ↑	0.026	0.014
anterior cingulate ctx	0.218	0.029	0.180 ↓	0.027	0.018
infralimbic ctx	0.219	0.039	0.175 ↓	0.032	0.029
agranular insular ctx	0.168	0.016	0.191 ↑	0.024	0.033
basal amygdala	0.203	0.014	0.182 ↓	0.020	0.035
medial amygdala	0.294	0.042	0.362 ↑	0.072	0.036
medial geniculate	0.195	0.023	0.217 ↑	0.017	0.040
posterior amygdala	0.273	0.090	0.388 ↑	0.116	0.042

Measures of radial diffusivity (RD) following lateral fluid percussion injury to the rostral (n = 5) and caudal (n = 8) cortex. Statistical differences between 150 brain regions comparing RD values between the affected ipsilateral side (right) and the contralateral side (left) are rank ordered for significance. Values are presented as the mean and standard deviation (SD). Areas underlined are common to both sites of cortical injury. Arrows denote the direction of the difference between the affected and control sides. Note that there is predominately an increase in RD with a few exceptions e.g., laterodorsal thalamus, basal amygdala, infralimbic ctx.

**Table 3:** Axial Diffusivity.

Right Rostral Cortical Insult—AD Values					
Region of Interest(ROI)	contralateral (left)		ipsilateral (right)		
	AD values	SD	AD values	SD	t-test
central amygdala	1.098	0.074	1.334 ↑	0.088	0.002
laterodorsal thalamus	1.322	0.149	0.988 ↓	0.099	0.003
lateral amygdala	1.136	0.044	1.360 ↑	0.129	0.006
raphe dorsal	1.650	0.139	1.968 ↑	0.199	0.019
medial amygdala	1.262	0.101	1.420 ↑	0.068	0.020
periaqueductal gray midbrain	1.054	0.063	1.188 ↑	0.091	0.027
medial geniculate	0.968	0.085	1.074 ↑	0.023	0.028
mediodorsal thalamus	1.240	0.063	1.340 ↑	0.071	0.046
Right Caudal Cortical Insult—AD Values					
Region of Interest(ROI)	contralateral (left)		ipsilateral (right)		
	AD values	SD	AD values	SD	t-test
central amygdala	1.071	0.025	1.221 ↑	0.088	0.001
mediodorsal thalamus	1.164	0.058	1.280 ↑	0.061	0.002
medial amygdala	1.204	0.040	1.327 ↑	0.086	0.002
dentate gyrus hippocampus	1.101	0.054	1.235 ↑	0.089	0.003
laterodorsal thalamus	1.410	0.162	1.172 ↓	0.107	0.004
subiculum hippocampus	1.088	0.027	1.195 ↑	0.085	0.004
globus pallidus	1.031	0.060	1.186 ↑	0.114	0.004
periaqueductal gray midbrain	1.165	0.073	1.300 ↑	0.105	0.010
lateral posterior thalamus	0.990	0.079	1.101 ↑	0.072	0.011
tenia tecta ctx	1.178	0.135	1.403 ↑	0.212	0.023
cortical amygdala	1.055	0.076	1.217 ↑	0.165	0.024
paraventricular n. hypo	1.029	0.087	1.167 ↑	0.155	0.045

Measures of axial diffusivity (AD) following fluid percussion injury to the rostal (n = 5) and caudal (n = 8) cortex. Statistical differences between 150 brain regions comparing AD values between the affected ipsilateral side (right) and the contralateral side (left) are rank ordered for significance. Values are presented as the mean and standard deviation (SD). Areas underlined are common to both sites of cortical injury. Arrows denote the direction of the difference between the affected and control sides. Note that there is predominately an increase in AD with only one exception the laterodorsal thalamus. These data from rostral and caudal concussions were collected 5 days post TBI.

Table 2 shows the comparison in RD values between the concussed (ipsilateral) and non-concussed (contralateral) sides of the brain for different regions of interest, rank ordered for their significance. RD values for rostral and caudal concussions are shown. While the general trend in these brain areas is for an increase in RD on the concussed ipsilateral side of the brain, there are again a few exceptions, e.g., paraventricular nucleus and laterodorsal thalamus, lateral geniculate, basal amygdala, and anterior cingulate and infralimbic cortices. In most cases these are the same areas identified in Table 1 for the FA values.

Table 3 shows the comparison in AD values between the concussed (ipsilateral) and non-concussed (contralateral) sides of the brain for different regions of interest, also rank ordered for their significance. AD values for rostral and caudal concussions are shown. While the general trend in these brain areas is an increase in AD on the concussed ipsilateral side of the brain, the only exception is the laterodorsal thalamus, which shows a decrease in AD values, a finding common across all IA values for both rostral and caudal concussions.

**Fig 2:**
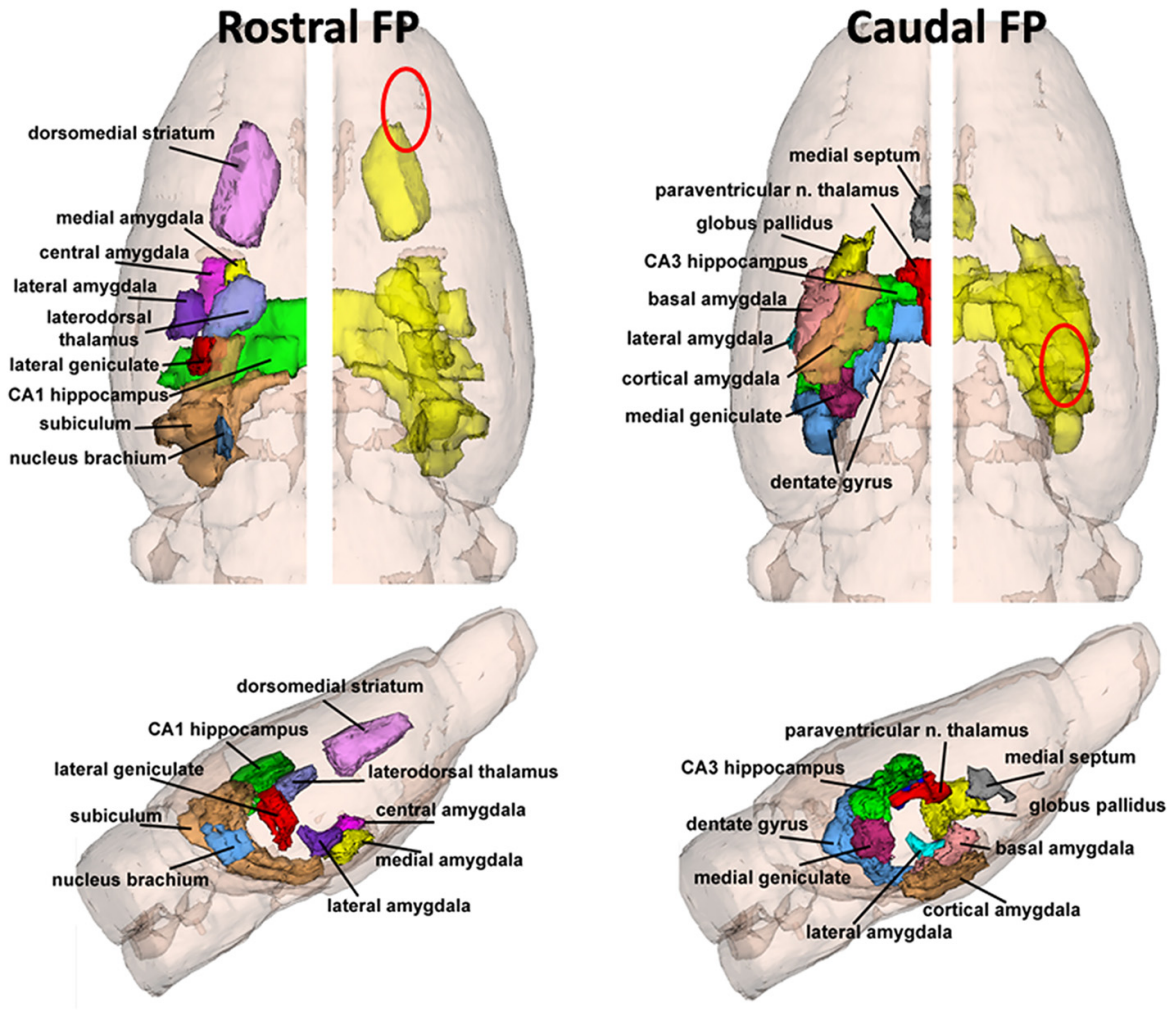
3D Reconstructions of Subcortical Brain Areas Sensitive to Cortical Contusion. The brain areas in Table 1 with significant differences in FA values between the ipsilateral concussed side of the brain and the contralateral side are shown in colored 3D volumes for both the rostral and the caudal fluid percussion injuries. Top images are coronal displays while the bottom images show a sagittal view of the brain. The individual brain areas are coalesced into a single volume (yellow) on the right side of the brain. The red oval depicts the approximate location of the fluid percussion injury. The significantly different cortical areas reported in Table 1 are not shown because they would obscure the visualization of the underlying subcortical brain areas. The medial amygdala and posterior amygdala from the Table 1 caudal concussion are not shown because they are hidden amongst the other amygdaloid nuclei.

Fig 2 shows a 3D reconstruction of the major subcortical areas involved in rostral and caudal concussions. The red ovals show the approximate locations of the concussion sites. The subcortical areas are highly represented by the amygdaloid complex, e.g., medial, cortical, lateral, basal and central amygdala; hippocampus, e.g., subiculum, CA1, CA3, and dentate gyrus; and thalamus, e.g., laterodorsal and paraventricular thalamic nuclei, and medial and lateral geniculate. The individual 3D volumes of each area are shown in color on the left, and coalesced into a single volume (yellow) on the right. This 3D perspective of affected areas in the context of the whole brain shows a common organization of subcortical sites vulnerable to FPI from different areas of the cortex. These common brain areas are very similar, often contiguous, and in some cases identical, e.g., central, medial and lateral nuclei amygdala and laterodorsal nucleus of the thalamus, as shown in blue in Tables 1–3.

**Fig 3:**
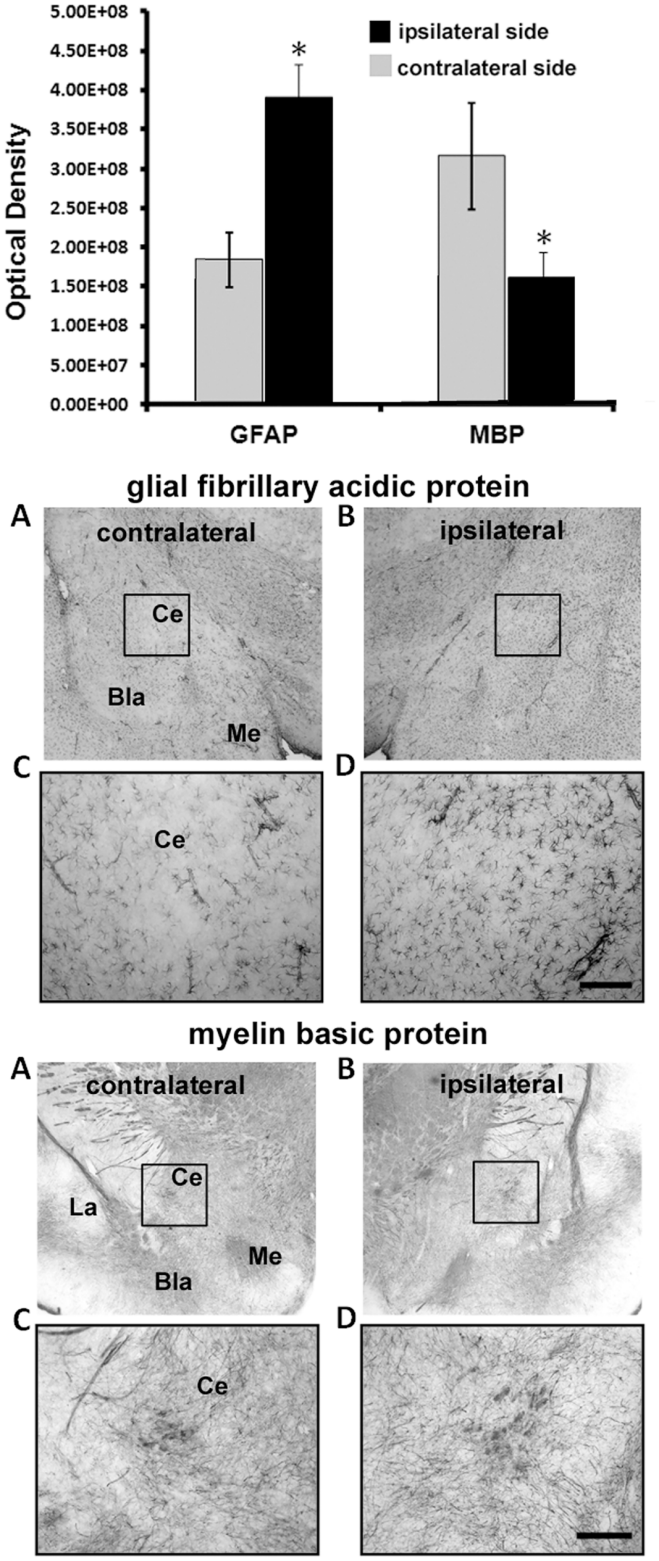
Injury to the Amygdala. Shown in the upper bar graphs are the comparisons in optical density (mean ± SE) of immunostaining for glial fibrillary acidic protein (GFAP) and myelin basic protein (MBP) between ipsilateral and contralateral sides of the amygdala. Photomicrographs of immunostaining are presented in the panels below for each molecular marker. Optical density for each was measured in the box sampling the area of the central nucleus of the amygdala (CE) depicted in photomicrographs A and B. Higher magnifications of the same areas are shown in photomicrographs C and D. The scale bare = 100 μm. * < 0.05; Abbreviations: Bla—basolateral amygdala; Me—medial amygdala; La—lateral amygdala.

**Fig 4:**
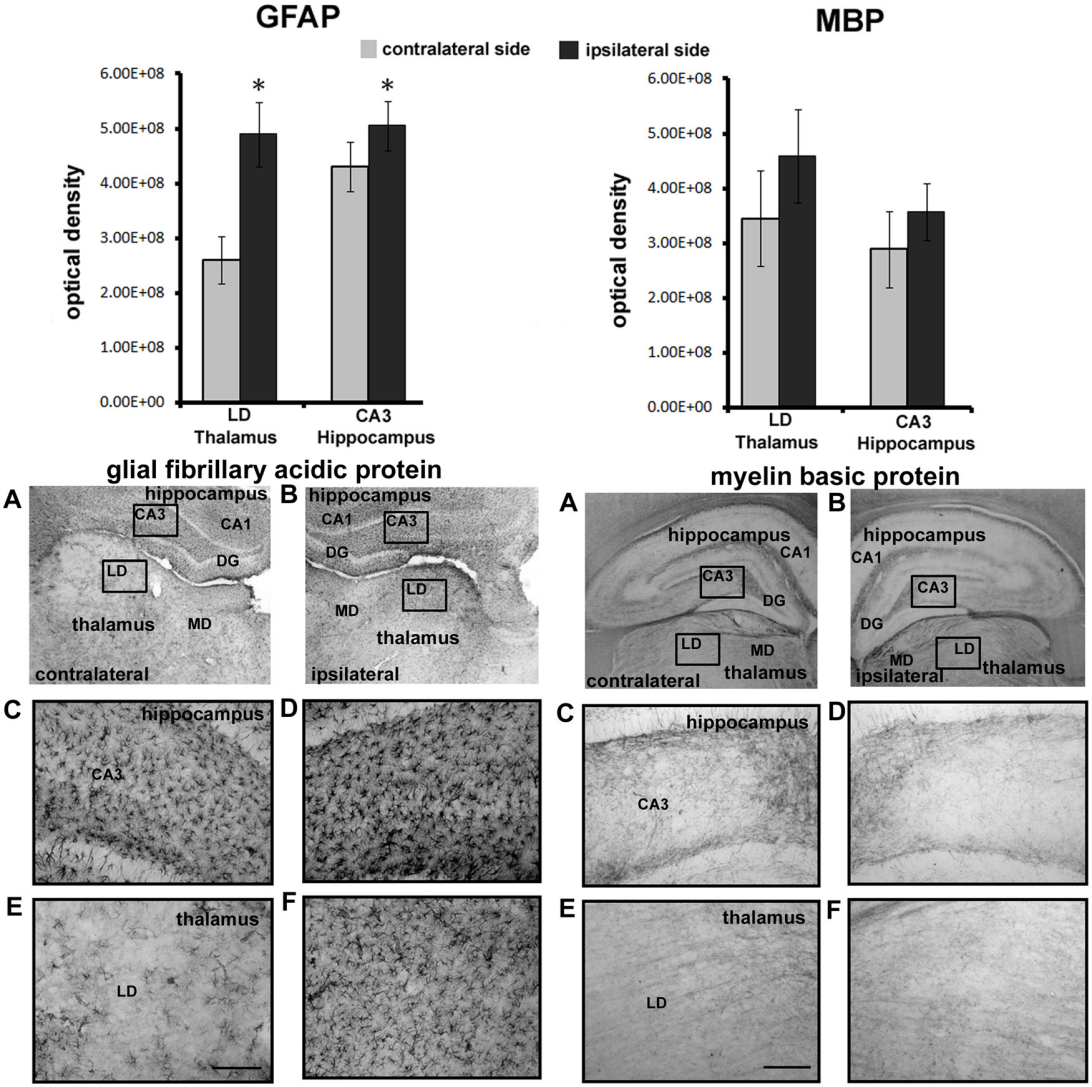
Injury to Thalamus and Hippocampus. Shown in the upper bar graphs are the comparisons in optical density (mean ± SE) of immunostaining for glial fibrillary acidic protein (GFAP) and myelin basic protein (MBP) between ipsilateral and contralateral sides of the thalamus and hippocampus. Photomicrographs of immunostaining are presented in the panels below for each molecular marker. Optical density for each was measured in the boxes sampling the areas of the laterodorsal thalamus (LD) and CA3 of the hippocampus depicted in photomicrographs A and B. Higher magnifications of the same areas are shown in photomicrographs C, D, E, and F. The scale bare = 100 μm. * < 0.05; Abbreviations: DG—dentate gyrus; CA1 area of the hippocampus; MD—mediodorsal thalamus.

Areas in the amygdala, thalamus and hippocampus showing common changes in IA values in response to both rostral and caudal FPI were analyzed with immunohistochemistry for changes in GFAP and MBP as measure of inflammation and axonal myelination, respectively. The photomicrographs in the top panel of Fig 3 show GFAP staining in the ipsilateral and contralateral central nucleus of the amygdala. Analyses of optical density revealed a greater than two-fold increase (p = 0.012) in immunostaining for GFAP in the concussed, ipsilateral central nucleus of the amygdala as compared to the contralateral side (bar graphs above), which suggests inflammatory processes occurring in the ipsilateral amygdala. The bottom panel of photomicrographs shows staining for MBP on the ipsilateral and contralateral central nucleus of the amygdala. There was a significant (p = 0.015) reduction in MBP levels on the ipsilateral, as compared to contralateral nucleus (bar graphs above), which suggests loss of myelinated fibers in this area of the amygdala. Shown in Fig 4 are GFAP and MBP staining and optical density values for the laterodorsal thalamus and CA3 hippocampus. The level of GFAP staining was significantly higher (LD p = 0.010; CA3 p = 0.038) in both brain areas on the affected ipsilateral side, as compared to the contralateral side (bar graphs above), which suggests the presence of inflammatory processes. There were no significant differences in MBP levels between contralateral and ipsilateral sides of the brain in the laterodorsal thalamus and CA3 hippocampus.

## Discussion

In this study we demonstrate for the first time that fluid percussion at two sites on the cortical mantel share common subcortical regions showing significant differences in diffusivity from the contralateral side of the brain. The gray matter, subcortical brain areas most sensitive to concussions at disparate sites on the cortex can be parsed into three major regions—the hippocampus, thalamus and the amygdala. This was demonstrated by changes in FA, RD and AD in discrete brain areas in each of these three regions on the ipsilateral side using DTI methodology. While, there was a small amount of edema at the points of contusion, they did not introduce any global deformation in the brain that could have influenced these changes. To confirm that these discrete brain areas identified *in vivo* with DTI were affected by cortical injury, we followed up with histological preparations to show neuroinflammation in all three affected brain regions and specific axonal demyelination in the central nucleus of the amygdala. Since these data were obtained using a new analytical method combining DTI maps registered into a 3D segmented rat atlas, we follow up below whether these findings are in agreement with those reported in the preclinical and clinical literature.

Studies on rodents using a variety of cellular and molecular markers to localize gray matter subcortical areas affected by different models of TBI all identify hippocampal involvement [11, 20–31]. Neuroinflammation measured by astrocyte expressing GFAP, as applied in this study, is routinely used as a cellular biomarker to follow disease progression post-concussion [25, 32]. Using stereological methods to quantify neuronal loss and glial proliferation, Grady and co-workers showed the hippocampus was selectively vulnerable to FPI given laterally or midline on the cortex [33]. The hilus was particularly sensitive to both sites of concussion, a finding corroborated in this study. In a multimodal imaging study Liu and colleagues followed developmental changes in the hippocampus following FPI [34]. PET imaging with (18)F-FDG revealed hypometabolism of the hippocampus that persisted for up to one month. There are many studies in humans using MRI based volumetric analysis or DTI that report a progressive reduction in hippocampal volume or alterations in IA values following TBI (for review see [11]. Hence preclinical and clinical studies show the hippocampus is vulnerable to the deleterious effects of TBI. This vulnerability of the hippocampus observed in our studies is consistent with the preclinical and clinical studies showing memory impairment following TBI [30, 31, 35–38]. Despite the wide use of DTI in clinical setting, there have been few animal studies reporting hippocampal changes as consequence to mild and moderate TBI. Most recently, studies in blast-induced TBI have reported structural changes in rat hippocampus using *ex-vivo* DTI [39, 40]. In the present study, assessment of gray matter damage in the hippocampus was initially made *in vivo* and later confirmed by postmortem histology showing neuroinflammation with increased GFAP levels.

Again, studies in rodents using different markers of neuroinflammation and neuronal damage have identified the thalamus as susceptible to the effects of TBI [22, 24, 25, 31, 41, 42]. Raghavendra and colleagues reported a time-dependent increase in microglia and astrocyte activation concomitant with neural death in the thalamus of the rat 1–14 days post-concussion [42]. The thalamus shows delayed yet persistent apoptosis beginning 1–2 weeks post FPI and continues for up to one month [22, 41]. Interestingly, in a recent study Das and coworkers reported that TBI initiates a systemic inflammatory reaction that precedes the local brain response and suggests that spleen- and/ or thymus-derived proinflammatory chemokines may promote and sustain neuronal injury in thalamus and hippocampus [22].

Patients recovering from TBI show significant thalamic hypometabolism [43]. It is common for patients with TBI to show progressive loss of thalamic volume [36, 44–47]. Reduction in thalamic volume and alterations in DTI are correlated with changes in cognitive function [36, 48, 49]. Little and colleagues have proposed a "thalamic hypothesis" as a central mechanism for the decrease in cognitive function following TBI [48]. Loss of connectivity between the forebrain cortex and the thalamic nuclei, particularly the dorsomedial nucleus, can affect executive function [50]. Indeed, the suppression of cortical oscillatory activity following TBI observed in the fluid percussion model suggests an injury-induced functional disruption of thalamocortical networks [51]. It should be noted that using DTI, the present study showed changes to the medial dorsal and laterodorsal thalamic nuclei in response to both rostral and caudal contusions. These findings are consistent with the clinical data and support the notion of thalamic vulnerability to diffuse TBI. Moreover, the ability to register DTI maps into the 3D segmented rat atlas allows one to identify in vivo damage to specific thalamic nuclei which can then be used as seed points in functional connectivity studies in the same animal.

Another subcortical area that appears vulnerable to TBI is the amygdala. In our studies we noted the central nucleus of the amygdala was affected by rostral and caudal contusions across several IA values (see Tables 1–3). In general it could be said that the amygdaloid complex was at risk since several nuclei, e.g., medial, lateral, and cortical were affected with one or both concussion sites. Interestingly, as compared to the hippocampus and thalamus, there is a paucity of literature on the amygdala in human TBI studies [36, 52]. This deficiency may be due to more subtle and more variable changes in gray matter volume in the amygdala following injury.

In preclinical studies, Hogg and coworkers [53] found no evidence of neurological deficits but did report a modest number of behavioral deficits in mild TBI caused by FPI. Rats with concussions localized to the parietal cortex show reduced contextual freezing suggesting a cognitive deficit in learning and memory involved in the formation of associations. In a subsequent study from the same laboratory, the brain was mapped with immunostaining for several inducible transcription factors to identify areas affected by mild TBI that could affect conditioned fear [21]. The hippocampus and the amygdaloid complex, particularly the central nucleus of the amygdala, showed significant differences from controls. In a more recent study, Rodgers and coworkers found increased reactive gliosis in the amygdala, particularly the basolateral and central nucleus in rats following FPI associated with exaggerated freezing behavior in a novel environment [32]. Our results corroborate these findings but open the field of TBI to an *in vivo* approach to identify discrete gray matter areas of neuropathology prior to post-mortem histology; thus making it possible to perform longitudinal studies on the same animal for the evaluation of new treatment strategies. Again the predictions made *in vivo* of gray matter damage to discrete amygdaloid areas was confirmed by postmortem histology showing neuroinflammmation by GFAP staining. It should be noted that the central nucleus of the amygdala also showed a decrease in MBP, suggesting injury to myelinated nerve fibers in and around the area. Indeed, the boundaries of the central nucleus of the amygdala encompass the commissural stria terminalis [54], a white matter tract originating from the lateral olfactory nucleus and the bed nucleus of the stria terminalis with projections to the anterior commissure [55].

While the neurological problems (e.g., motor and visual disturbances) associated with mild to moderate TBI may recover soon after insult [56], problems involving dysregulation of emotion and cognition may persist for months or even years [57, 58]. Indeed, changes in mood and anxiety are common psychiatric disorders following TBI. It has been hypothesized that dysregulation of prefrontal control over limbic cortex and amygdala may underlie these changes in behavior following concussion. The discovery in this study that the amygdala, hippocampus and dorsal thalamic nuclei are vulnerable to modest diffuse fluid percussion is particularly relevant to the clinical perspective on TBI. Connections between the hippocampus and amygdala [59, 60] and those between dorsal thalamic nuclei and limbic cortex [61] provide a frame work of distributed neural circuitry affecting behavior, learning and memory [62]. The sensitivity of the central nucleus of the amygdala to diffuse cortical impacts may be key to understanding the psychiatric consequence, like depression and post-traumatic stress disorder (PTSD) that are strongly associated with mild TBI. The models of PTSD have the amygdala and its connections to the hippocampus and prefrontal cortex as key neural substrates contributing to the exaggerated fear, anxiety and altered stress [63]. Major depression is a frequent complication of TBI and is associated with executive dysfunction, negative affect, and prominent anxiety symptoms [64].

## References

[1] Shenton ME, Hamoda HM, Schneiderman JS, Bouix S, Pasternak O, Rathi Y, et al A review of magnetic resonance imaging and diffusion tensor imaging findings in mild traumatic brain injury. Brain imaging and behavior. 2012;6(2):137–92. Epub 2012/03/23. 10.1007/s11682-012-9156-5 . 22438191 PMC3803157

[2] Messe A, Caplain S, Paradot G, Garrigue D, Mineo JF, Soto Ares G, et al Diffusion tensor imaging and white matter lesions at the subacute stage in mild traumatic brain injury with persistent neurobehavioral impairment. Human brain mapping. 2011;32(6):999–1011. Epub 2010/07/30. 10.1002/hbm.21092 . 20669166 PMC6870077

[3] Brezova V, K GRM, Skandsen T, Vik A, Brewer JB, Salvesen O, et al Prospective longitudinal MRI study of brain volumes and diffusion changes during the first year after moderate to severe traumatic brain injury. Neuroimage Clin. 2014;5:128–40. 10.1016/j.nicl.2014.03.012 25068105 PMC4110353

[4] Rathi Y, Pasternak O, Savadjiev P, Michailovich O, Bouix S, Kubicki M, et al Gray matter alterations in early aging: A diffusion magnetic resonance imaging study. Hum Brain Mapp. 2014;35(8):3841–56. 10.1002/hbm.22441 24382651 PMC4101075

[5] Pasternak O, Westin CF, Dahlben B, Bouix S, Kubicki M. The extent of diffusion MRI markers of neuroinflammation and white matter deterioration in chronic schizophrenia. Schizophrenia research. 2014 Epub 2014/08/16. 10.1016/j.schres.2014.07.031 . 25126717 PMC4277709

[6] Kou Z, Wu Z, Tong KA, Holshouser B, Benson RR, Hu J, et al The role of advanced MR imaging findings as biomarkers of traumatic brain injury. The Journal of head trauma rehabilitation. 2010;25(4):267–82. Epub 2010/07/09. 10.1097/HTR.0b013e3181e54793 . 20611045

[7] Bazarian JJ, Zhu T, Blyth B, Borrino A, Zhong J. Subject-specific changes in brain white matter on diffusion tensor imaging after sports-related concussion. Magn Reson Imaging. 2012;30(2):171–80. Epub 2011/11/15. 10.1016/j.mri.2011.10.001 22079073 PMC3254806

[8] Bozzali M, Cercignani M, Sormani MP, Comi G, Filippi M. Quantification of brain gray matter damage in different MS phenotypes by use of diffusion tensor MR imaging. Ajnr. 2002;23(6):985–8. . 12063230 PMC7976912

[9] Bouix S, Pasternak O, Rathi Y, Pelavin PE, Zafonte R, Shenton ME. Increased gray matter diffusion anisotropy in patients with persistent post-concussive symptoms following mild traumatic brain injury. PLoS One. 2013;8(6):e66205 10.1371/journal.pone.0066205 23776631 PMC3679020

[10] Budde MD, Janes L, Gold E, Turtzo LC, Frank JA. The contribution of gliosis to diffusion tensor anisotropy and tractography following traumatic brain injury: validation in the rat using Fourier analysis of stained tissue sections. Brain. 2011;134(Pt 8):2248–60. 10.1093/brain/awr161 21764818 PMC3155707

[11] McIntosh TK, Vink R, Noble L, Yamakami I, Fernyak S, Soares H, et al Traumatic brain injury in the rat: characterization of a lateral fluid-percussion model. Neuroscience. 1989;28(1):233–44. Epub 1989/01/01. . 2761692 10.1016/0306-4522(89)90247-9

[12] Kharatishvili I, Nissinen JP, McIntosh TK, Pitkanen A. A model of posttraumatic epilepsy induced by lateral fluid-percussion brain injury in rats. Neuroscience. 2006;140(2):685–97. Epub 2006/05/03. 10.1016/j.neuroscience.2006.03.012 . 16650603

[13] Johnsen EL, Tranel D, Lutgendorf S, Adolphs R. A neuroanatomical dissociation for emotion induced by music. International journal of psychophysiology: official journal of the International Organization of Psychophysiology. 2009;72(1):24–33. Epub 2008/10/01. 10.1016/j.ijpsycho.2008.03.011 18824047 PMC2656600

[14] Moseley ME, Kucharczyk J, Asgari HS, Norman D. Anisotropy in diffusion-weighted MRI. Magn Reson Med. 1991;19(2):321–6. Epub 1991/06/01. . 1652674 10.1002/mrm.1910190222

[15] Song SK, Sun SW, Ju WK, Lin SJ, Cross AH, Neufeld AH. Diffusion tensor imaging detects and differentiates axon and myelin degeneration in mouse optic nerve after retinal ischemia. NeuroImage. 2003;20(3):1714–22. Epub 2003/12/04. . 14642481 10.1016/j.neuroimage.2003.07.005

[16] McCloskey MS, Lee R, Berman ME, Noblett KL, Coccaro EF. The relationship between impulsive verbal aggression and intermittent explosive disorder. Aggressive behavior. 2008;34(1):51–60. Epub 2007/07/27. 10.1002/ab.20216 . 17654692

[17] McCloskey MS, Ben-Zeev D, Lee R, Berman ME, Coccaro EF. Acute tryptophan depletion and self-injurious behavior in aggressive patients and healthy volunteers. Psychopharmacology (Berl). 2009;203(1):53–61. Epub 2008/10/24. 10.1007/s00213-008-1374-6 . 18946662

[18] Coccaro EF, Bergeman CS, Kavoussi RJ, Seroczynski AD. Heritability of aggression and irritability: a twin study of the Buss-Durkee aggression scales in adult male subjects. Biol Psychiatry. 1997;41(3):273–84. Epub 1997/02/01. . 9024950 10.1016/s0006-3223(96)00257-0

[19] Coccaro EF, Kavoussi RJ, Sheline YI, Berman ME, Csernansky JG. Impulsive aggression in personality disorder correlates with platelet 5-HT2A receptor binding. Neuropsychopharmacology. 1997;16(3):211–6. Epub 1997/03/01. 10.1016/S0893-133X(96)00194-7 . 9138437

[20] Raghupathi R, McIntosh TK. Regionally and temporally distinct patterns of induction of c-fos, c-jun and junB mRNAs following experimental brain injury in the rat. Brain Res Mol Brain Res. 1996;37(1–2):134–44. Epub 1996/04/01. . 8738144 10.1016/0169-328x(95)00289-5

[21] Abrous DN, Rodriguez J, le Moal M, Moser PC, Barneoud P. Effects of mild traumatic brain injury on immunoreactivity for the inducible transcription factors c-Fos, c-Jun, JunB, and Krox-24 in cerebral regions associated with conditioned fear responding. Brain Res. 1999;826(2):181–92. Epub 1999/05/04. . 10224295 10.1016/s0006-8993(99)01259-7

[22] Das M, Leonardo CC, Rangooni S, Pennypacker KR, Mohapatra S, Mohapatra SS. Lateral fluid percussion injury of the brain induces CCL20 inflammatory chemokine expression in rats. Journal of neuroinflammation. 2011;8:148 Epub 2011/11/02. 10.1186/1742-2094-8-148 22040257 PMC3231817

[23] Hayes RL, Yang K, Raghupathi R, McIntosh TK. Changes in gene expression following traumatic brain injury in the rat. Journal of neurotrauma. 1995;12(5):779–90. Epub 1995/10/01. . 8594207 10.1089/neu.1995.12.779

[24] Hicks R, Soares H, Smith D, McIntosh T. Temporal and spatial characterization of neuronal injury following lateral fluid-percussion brain injury in the rat. Acta neuropathologica. 1996;91(3):236–46. Epub 1996/01/01. . 8834535 10.1007/s004010050421

[25] Hill SJ, Barbarese E, McIntosh TK. Regional heterogeneity in the response of astrocytes following traumatic brain injury in the adult rat. J Neuropathol Exp Neurol. 1996;55(12):1221–9. Epub 1996/12/01. . 8957445 10.1097/00005072-199612000-00005

[26] Hovda DA, Yoshino A, Kawamata T, Katayama Y, Becker DP. Diffuse prolonged depression of cerebral oxidative metabolism following concussive brain injury in the rat: a cytochrome oxidase histochemistry study. Brain Res. 1991;567(1):1–10. Epub 1991/12/13. . 1667742 10.1016/0006-8993(91)91429-5

[27] Nonaka M, Chen XH, Pierce JE, Leoni MJ, McIntosh TK, Wolf JA, et al Prolonged activation of NF-kappaB following traumatic brain injury in rats. Journal of neurotrauma. 1999;16(11):1023–34. Epub 1999/12/14. . 10595819 10.1089/neu.1999.16.1023

[28] Yoshino A, Hovda DA, Kawamata T, Katayama Y, Becker DP. Dynamic changes in local cerebral glucose utilization following cerebral conclusion in rats: evidence of a hyper- and subsequent hypometabolic state. Brain Res. 1991;561(1):106–19. Epub 1991/10/04. . 1797338 10.1016/0006-8993(91)90755-k

[29] Yu I, Inaji M, Maeda J, Okauchi T, Nariai T, Ohno K, et al Glial cell-mediated deterioration and repair of the nervous system after traumatic brain injury in a rat model as assessed by positron emission tomography. Journal of neurotrauma. 2010;27(8):1463–75. Epub 2010/05/28. 10.1089/neu.2009.1196 . 20504160

[30] Hicks RR, Smith DH, Lowenstein DH, Saint Marie R, McIntosh TK. Mild experimental brain injury in the rat induces cognitive deficits associated with regional neuronal loss in the hippocampus. Journal of neurotrauma. 1993;10(4):405–14. Epub 1993/01/01. . 8145264 10.1089/neu.1993.10.405

[31] Hylin MJ, Orsi SA, Zhao J, Bockhorst K, Perez A, Moore AN, et al Behavioral and histopathological alterations resulting from mild fluid percussion injury. Journal of neurotrauma. 2013;30(9):702–15. Epub 2013/01/11. 10.1089/neu.2012.2630 . 23301501 PMC3941923

[32] Rodgers KM, Bercum FM, McCallum DL, Rudy JW, Frey LC, Johnson KW, et al Acute neuroimmune modulation attenuates the development of anxiety-like freezing behavior in an animal model of traumatic brain injury. Journal of neurotrauma. 2012;29(10):1886–97. Epub 2012/03/23. 10.1089/neu.2011.2273 22435644 PMC3390983

[33] Grady MS, Charleston JS, Maris D, Witgen BM, Lifshitz J. Neuronal and glial cell number in the hippocampus after experimental traumatic brain injury: analysis by stereological estimation. Journal of neurotrauma. 2003;20(10):929–41. Epub 2003/11/01. 10.1089/089771503770195786 . 14588110

[34] Liu YR, Cardamone L, Hogan RE, Gregoire MC, Williams JP, Hicks RJ, et al Progressive metabolic and structural cerebral perturbations after traumatic brain injury: an in vivo imaging study in the rat. J Nucl Med. 2010;51(11):1788–95. Epub 2010/11/06. 10.2967/jnumed.110.078626 . 21051651

[35] Umile EM, Sandel ME, Alavi A, Terry CM, Plotkin RC. Dynamic imaging in mild traumatic brain injury: support for the theory of medial temporal vulnerability. Archives of physical medicine and rehabilitation. 2002;83(11):1506–13. Epub 2002/11/08. . 12422317 10.1053/apmr.2002.35092

[36] Warner MA, Youn TS, Davis T, Chandra A, Marquez de la Plata C, Moore C, et al Regionally selective atrophy after traumatic axonal injury. Arch Neurol. 2010;67(11):1336–44. Epub 2010/07/14. 10.1001/archneurol.2010.149 20625067 PMC3465162

[37] Himanen L, Portin R, Isoniemi H, Helenius H, Kurki T, Tenovuo O. Cognitive functions in relation to MRI findings 30 years after traumatic brain injury. Brain injury: [BI]. 2005;19(2):93–100. Epub 2005/04/22. . 15841753 10.1080/02699050410001720031

[38] Tate DF, Bigler ED. Fornix and hippocampal atrophy in traumatic brain injury. Learn Mem. 2000;7(6):442–6. Epub 2000/12/12. . 11112803 10.1101/lm.33000

[39] Gussin HA, Tomlinson ID, Muni NJ, Little DM, Qian H, Rosenthal SJ, et al GABAC receptor binding of quantum-dot conjugates of variable ligand valency. Bioconjugate chemistry. 2010;21(8):1455–64. Epub 2010/08/19. 10.1021/bc100050s 20715850 PMC2929923

[40] Silverstein SM, Berten S, Essex B, All SD, Kasi R, Little DM. Perceptual organization and visual search processes during target detection task performance in schizophrenia, as revealed by fMRI. Neuropsychologia. 2010;48(10):2886–93. Epub 2010/08/04. 10.1016/j.neuropsychologia.2010.05.030 . 20678981

[41] Conti AC, Raghupathi R, Trojanowski JQ, McIntosh TK. Experimental brain injury induces regionally distinct apoptosis during the acute and delayed post-traumatic period. J Neurosci. 1998;18(15):5663–72. Epub 1998/07/22. . 9671657 10.1523/JNEUROSCI.18-15-05663.1998 PMC6793063

[42] Raghavendra Rao VL, Dogan A, Bowen KK, Dempsey RJ. Traumatic brain injury leads to increased expression of peripheral-type benzodiazepine receptors, neuronal death, and activation of astrocytes and microglia in rat thalamus. Exp Neurol. 2000;161(1):102–14. Epub 2000/02/23. 10.1006/exnr.1999.7269 . 10683277

[43] Lull N, Noe E, Lull JJ, Garcia-Panach J, Chirivella J, Ferri J, et al Voxel-based statistical analysis of thalamic glucose metabolism in traumatic brain injury: relationship with consciousness and cognition. Brain injury: [BI]. 2010;24(9):1098–107. Epub 2010/07/06. 10.3109/02699052.2010.494592 . 20597637

[44] Anderson CV, Bigler ED, Blatter DD. Frontal lobe lesions, diffuse damage, and neuropsychological functioning in traumatic brain-injured patients. Journal of clinical and experimental neuropsychology. 1995;17(6):900–8. Epub 1995/12/01. 10.1080/01688639508402438 . 8847395

[45] Yount R, Raschke KA, Biru M, Tate DF, Miller MJ, Abildskov T, et al Traumatic brain injury and atrophy of the cingulate gyrus. J Neuropsychiatry Clin Neurosci. 2002;14(4):416–23. Epub 2002/11/12. . 12426409 10.1176/jnp.14.4.416

[46] Strangman GE, O'Neil-Pirozzi TM, Supelana C, Goldstein R, Katz DI, Glenn MB. Regional brain morphometry predicts memory rehabilitation outcome after traumatic brain injury. Frontiers in human neuroscience. 2010;4:182 Epub 2010/11/05. 10.3389/fnhum.2010.00182 21048895 PMC2967347

[47] Warner MA, Marquez de la Plata C, Spence J, Wang JY, Harper C, Moore C, et al Assessing spatial relationships between axonal integrity, regional brain volumes, and neuropsychological outcomes after traumatic axonal injury. Journal of neurotrauma. 2010;27(12):2121–30. Epub 2010/09/30. 10.1089/neu.2010.1429 20874032 PMC2996819

[48] Little DM, Kraus MF, Joseph J, Geary EK, Susmaras T, Zhou XJ, et al Thalamic integrity underlies executive dysfunction in traumatic brain injury. Neurology. 2010;74(7):558–64. Epub 2010/01/22. 10.1212/WNL.0b013e3181cff5d5 20089945 PMC2830915

[49] Grossman EJ, Ge Y, Jensen JH, Babb JS, Miles L, Reaume J, et al Thalamus and cognitive impairment in mild traumatic brain injury: a diffusional kurtosis imaging study. Journal of neurotrauma. 2012;29(13):2318–27. Epub 2011/06/07. 10.1089/neu.2011.1763 21639753 PMC3430483

[50] Geary EK, Kraus MF, Pliskin NH, Little DM. Verbal learning differences in chronic mild traumatic brain injury. Journal of the International Neuropsychological Society: JINS. 2010;16(3):506–16. Epub 2010/03/02. 10.1017/S135561771000010X . 20188015

[51] Kao C, Forbes JA, Jermakowicz WJ, Sun DA, Davis B, Zhu J, et al Suppression of thalamocortical oscillations following traumatic brain injury in rats. Journal of neurosurgery. 2012;117(2):316–23. Epub 2012/05/29. 10.3171/2012.4.JNS111170 . 22631688

[52] Wilde EA, Bigler ED, Hunter JV, Fearing MA, Scheibel RS, Newsome MR, et al Hippocampus, amygdala, and basal ganglia morphometrics in children after moderate-to-severe traumatic brain injury. Developmental medicine and child neurology. 2007;49(4):294–9. Epub 2007/03/23. 10.1111/j.1469-8749.2007.00294.x . 17376141

[53] Hogg S, Moser PC, Sanger DJ. Mild traumatic lesion of the right parietal cortex of the rat: selective behavioural deficits in the absence of neurological impairment. Behav Brain Res. 1998;93(1–2):143–55. Epub 1998/07/11. . 9659996 10.1016/s0166-4328(97)00146-0

[54] Paxinos G, Watson C. The rat brain in stereotaxic coordinates. Academic Press 1986. 10.1016/0165-0270(80)90021-7 6110810

[55] McCloskey MS, Ben-Zeev D, Lee R, Coccaro EF. Prevalence of suicidal and self-injurious behavior among subjects with intermittent explosive disorder. Psychiatry research. 2008;158(2):248–50. Epub 2008/01/29. 10.1016/j.psychres.2007.09.011 18221794 PMC2291349

[56] Binder LM. A review of mild head trauma. Part II: Clinical implications. Journal of clinical and experimental neuropsychology. 1997;19(3):432–57. Epub 1997/06/01. 10.1080/01688639708403871 . 9268817

[57] Millis SR, Rosenthal M, Novack TA, Sherer M, Nick TG, Kreutzer JS, et al Long-term neuropsychological outcome after traumatic brain injury. The Journal of head trauma rehabilitation. 2001;16(4):343–55. Epub 2001/07/20. . 11461657 10.1097/00001199-200108000-00005

[58] Whitnall L, McMillan TM, Murray GD, Teasdale GM. Disability in young people and adults after head injury: 5–7 year follow up of a prospective cohort study. Journal of neurology, neurosurgery, and psychiatry. 2006;77(5):640–5. Epub 2006/04/15. 10.1136/jnnp.2005.078246 16614025 PMC2117429

[59] Ishikawa A, Nakamura S. Ventral hippocampal neurons project axons simultaneously to the medial prefrontal cortex and amygdala in the rat. J Neurophysiol. 2006;96(4):2134–8. Epub 2006/07/14. 10.1152/jn.00069.2006 . 16837666

[60] Wang L, Mamah D, Harms MP, Karnik M, Price JL, Gado MH, et al Progressive deformation of deep brain nuclei and hippocampal-amygdala formation in schizophrenia. Biol Psychiatry. 2008;64(12):1060–8. Epub 2008/09/26. 10.1016/j.biopsych.2008.08.007 18814865 PMC2855119

[61] Wang JY, Bakhadirov K, Abdi H, Devous MD Sr., Marquez de la Plata CD, Moore C, et al Longitudinal changes of structural connectivity in traumatic axonal injury. Neurology. 2011;77(9):818–26. Epub 2011/08/05. 10.1212/WNL.0b013e31822c61d7 21813787 PMC3162636

[62] Shin LM, Liberzon I. The neurocircuitry of fear, stress, and anxiety disorders. Neuropsychopharmacology. 2010;35(1):169–91. Epub 2009/07/25. 10.1038/npp.2009.83 19625997 PMC3055419

[63] Rauch SL, Shin LM, Phelps EA. Neurocircuitry models of posttraumatic stress disorder and extinction: human neuroimaging research—past, present, and future. Biol Psychiatry. 2006;60(4):376–82. Epub 2006/08/22. 10.1016/j.biopsych.2006.06.004 . 16919525

[64] Maller JJ, Thomson RH, Lewis PM, Rose SE, Pannek K, Fitzgerald PB. Traumatic brain injury, major depression, and diffusion tensor imaging: making connections. Brain research reviews. 2010;64(1):213–40. Epub 2010/04/15. 10.1016/j.brainresrev.2010.04.003 . 20388528

